# Monte Carlo simulation of tilted contact plaque brachytherapy placement for juxtapapillary retinoblastoma

**DOI:** 10.1186/s13014-022-01986-8

**Published:** 2022-01-24

**Authors:** Satoshi Nakamura, Naoya Murakami, Shigenobu Suzuki, Kimiteru Ito, Mihiro Takemori, Hiroki Nakayama, Keita Kaga, Takahito Chiba, Kotaro Iijima, Kana Takahashi, Tomonori Goka, Jun Itami, Hiroyuki Okamoto, Hiroshi Igaki

**Affiliations:** 1 Department of Medical Physics, National Cancer Center Hospital, Tokyo, Japan; 2 Devision of Research and Development for Boron Neutron Capture Therapy, National Cancer Center Exploratory Oncology Research & Clinical Trial Center, Tokyo, Japan; 3 Department of Radiation Oncology, National Cancer Center Hospital, Tsukiji 5-1-1, Chuo-ku, Tokyo, 104-0045 Japan; 4 Department of Ophthalmic Oncology, National Cancer Center Hospital, Tokyo, Japan; 5 Department of Diagnostic Radiology, National Cancer Center Hospital, Tokyo, Japan; 6 Department of Radiological Technology, National Cancer Center Hospital, Tokyo, Japan; 7 Department of Radiological Science, Graduate School of Human Health Sciences, Higashi-ogu 7-2-10, Arakawa-ku, Tokyo 116-8551 Japan

**Keywords:** ^106^Ru plaque applicator, Brachytherapy, Retinoblastoma, Monte Carlo simulation, Juxtapapillary tumor

## Abstract

**Background:**

The 106-Ruthenium contact plaque applicator is utilized for the treatment of intraocular tumor within a thickness of less than 6 mm. If anything obstructs the placement of the plaque applicator, the treatment is generally difficult because the applicator has to be temporarily located just on the opposite side of the retinal tumor. Furthermore, the plaque applicator edge of approximately 1 mm does not contain ^106^Ru, estimating the delivered radiation dose for eccentric tumor is challenging because the lateral dose profile is inadequately provided by the manufacture’s certification. This study aims to simulate tumor coverage of the tilted applicator placement for treating an infant with juxtapapillary retinoblastoma and to achieve the effective treatment.

**Case presentation:**

We present an infant with retinoblastoma whose tumor involved macular and was invading just temporal side of the optic disc. Additionally, posterior staphyloma was induced by a series of previous treatments, making it more difficult to treat the standard plaque placement. Thus, the applicator type of CCA was intentionally tilted to the eyeball and the distance between the posterior edge of the applicator and the eyeball had to be then equal to or more than 2 mm based on the dose distribution of the applicator calculated using Monte Carlo simulation to minimize damage to surrounding tissues while covering the tumor. It was then comparable to the certification and previous reports. Based on the acquired dose distribution, the optimal placement of the applicator was derived from varying the distance between the applicator’s edge and the eyeball, and the distance was then determined to be 2 mm. In this case, the minimum dose rate in the tumor was 25.5 mGy/min, and the time required to deliver the prescribed dose was 26.2 h. Therefore, the tilted ^106^Ru plaque applicator placement could deliver the required dose for the treatment. The physical examination revealed no active tumor as a result of the treatment.

**Conclusions:**

Optimizing the placement of the ^106^Ru plaque applicator, it was possible to guarantee that the prescribed dose will be delivered to the tumor even if the standard placement is not possible for the juxtapapillary tumor.

## Background

Contact plaque brachytherapy has been shown to be effective in treating that early-stage retinoblastoma, with an overall survival rate comparable to enucleation. Because 106-Ruthenium (^106^Ru) is a pure beta emitter with a short range, intraocular tumors with a height of less than 6 mm are suitable for treatment with ^106^Ru contact plaque brachytherapy [1]. Another requirement for plaque brachytherapy is that the applicator be temporarily placed just on the opposite side of the retinal tumor. However, if anything obstructs the placement of the plaque applicator, plaque brachytherapy cannot be used to treat retinal tumors. Furthermore, because the plaque applicator edge of approximately 1 mm does not contain ^106^Ru, estimating the true radiation dose for eccentric tumors is challenging. Here, we present an infant with retinoblastoma whose tumor involved macular and spread just next to the optic disc, making standard plaque placement difficult. Additionally, the radii of curvature of the plaque applicators are designed between 12 and 14 mm because they are designed to treat adult patients, whereas the radius of infants’ eye is around 9 mm. As a result, if the applicator’s central part is attached to the eyeball, its edge will inevitably float, and portion of the tumor that will be covered by the applicator’s peripheral part will receive an inadequate radiation dose. If the portion of the tumor covered by the peripheral part of the plaque applicator is more vital than the central part, it may be appropriate to attach the edge of the applicator to the eyeball. Thus, an optimal placement method based on the dose distribution and applicator’s configuration is required to ensure the prescribed dose is delivered to the tumor. The manufacture certifies the depth dose distribution based on measurement results referring to the scintillator’s distance from the midpoint of the applicator’s inner surface. However, the off-axis delivered doses in only a few representative points are provided to evaluate the rough lateral dose profile. Therefore, the precise dose distribution of the applicator’s peripheral part is unknown. The treatment planning system (Plaque Simulator, Bebig) can generate three-dimensional dose distribution. However, the unusual placement of the applicator such as tilting the applicator against the eyeball is not possible to simulate the dose distribution in the treatment planning system. Thus, because the most concerning aspect of this case was dose coverage of the applicator’s peripheral parts, personalized dose calculation simulation was required. As a result, we selected the best plaque placement from a number of options by simulating the dose distribution derived from the Monte Carlo Simulation.

## Methods

### Case presentation

**Fig. 1:**
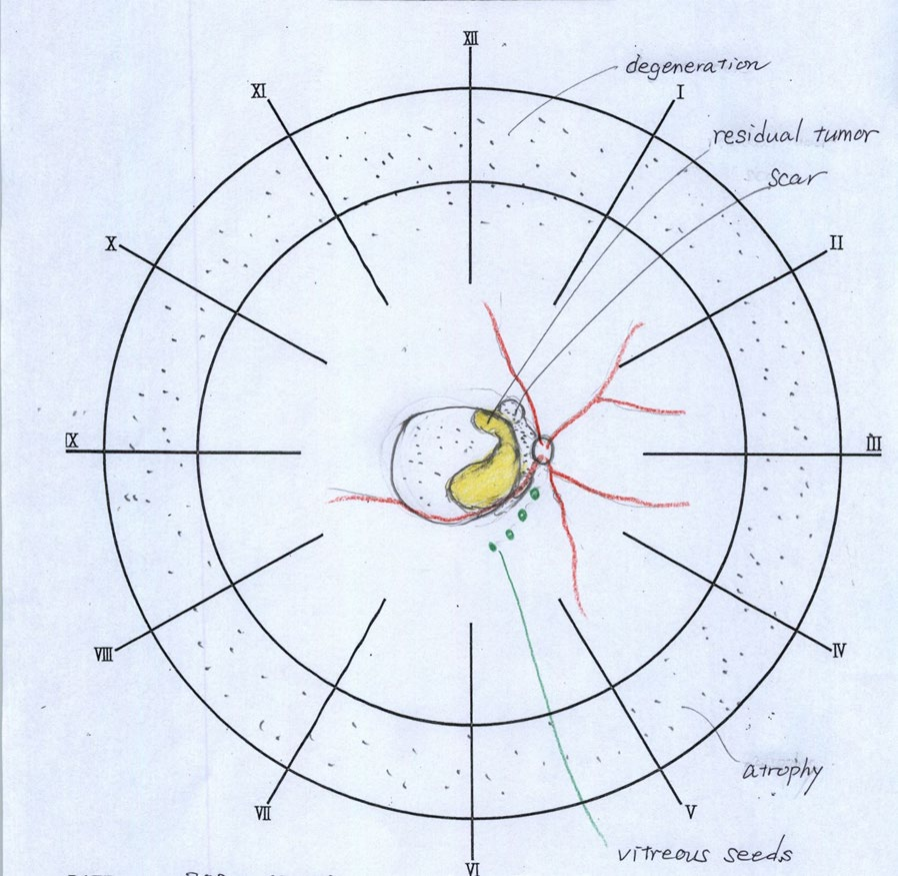
Schematic diagram for the relationships between the tumor and the optic disc

**Fig. 2:**
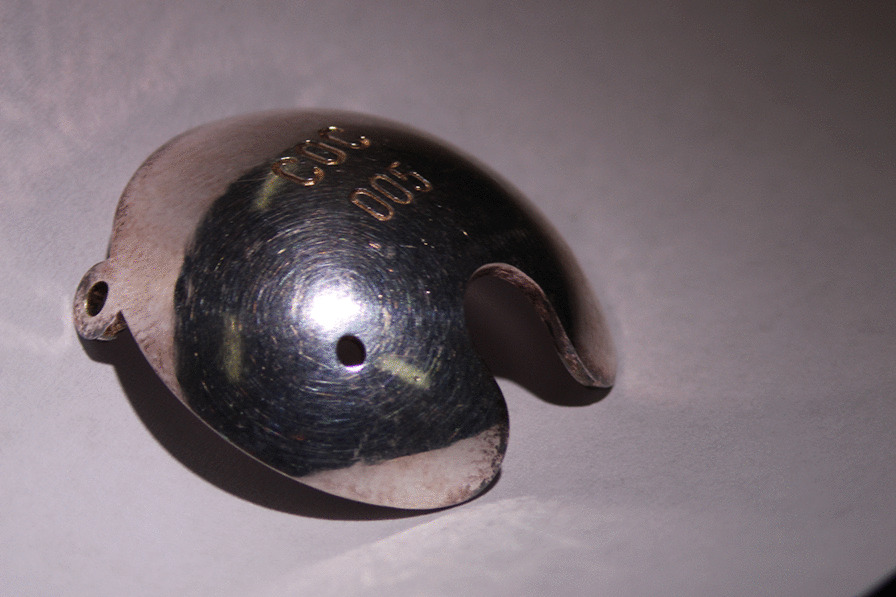
Picture of the COC type of the ^106^Ru applicator

**Fig. 3:**
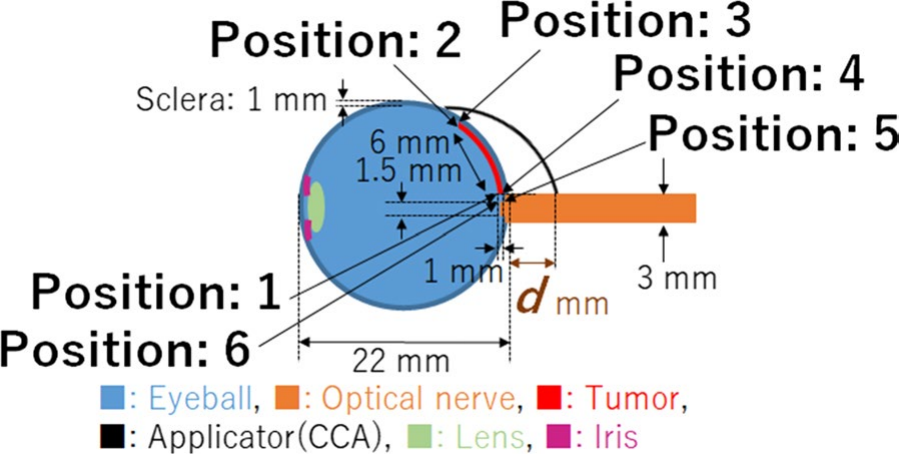
The schematic diagram for the horizontal plane in this case

A three-year old boy having no family history of retinoblastoma presented with bilateral retinoblastoma. The International Classification of Retinoblastoma (ICRB) group B in right eye and E in the left eye, respectively, and the tumor of his right eye was located posterior area involved macula and attached to the optic disc. After enucleation of the left eye, he received eight cycles of systemic chemotherapy with vincristine, etoposide, and carboplatin. Soon after the systemic chemotherapy, tumor regrowth at the macula was observed. Despite eight sessions of intra-arterial chemotherapy with melphalan and topotecan and two sessions of intra-vitreal chemotherapy injection with melphalan, the macular tumor persisted. Six more cycles of systemic chemotherapy with vincristine, doxorubicin, and cyclophosphamide, as well as proton beam radiotherapy was attempted, but the tumor remained active. Following additional intra-arterial chemotherapy, the boy was referred to our hospital for additional salvage therapy. As a salvage treatment, ^106^Ru plaque brachytherapy (Bebig GmbH, Berlin, Germany) was planned. Figure 1 depicts the clinical course of fundus photos. A residual tumor was found in the nasal half of the scar, invading the optic disc (Fig. 1). The COC type is a specific type of applicator designed to treat tumors near the optic nerve and has a deep concave to avoid the optic nerve (Fig. 2). However, because this patient’s tumor was on the temporal side of the optic disc (Fig. 1), placing the COC applicator was difficult and dangerous because the oblique muscles, posterior ciliary nerves, and posterior ciliary arteries could be damaged by placing the COC applicator. Therefore, CCA, a small round-shaped applicator, was chosen for this patient. Another disadvantage of this patient was that he had posterior staphyloma as a result of a series of previous treatments, making it more difficult to attach the plaque to the scleral surface and the distance (*d*) between the posterior edge of the applicator and the eyeball had to be at least 2 mm (Fig. 3). To adequately cover part of the tumor located adjacent to the optic disc, the CCA applicator was intentionally tilted to the eyeball. During operation, echography was used to determine the exact location of the plaque.

### Validation of calculated dose distribution

Monte Carlo simulation (Particle and Heavy Ion Transport code System (PHITS), ver. 2.820) [2] was used to calculate the dose distribution in water. The energy spectrum of the emitted beta ray from the applicator was calculated based on the International Commission on Radiation Units and Measurements (ICRU) report no. 72 [3]. In the simulation, the cut-off energy for a beta ray is set to 7.0 × 10^–2^ eV, and its range is water equivalent to 0.1 mm. Furthermore, the gamma ray emitted by daughter radionuclide decay (106-Rhodium) was also calculated. The gamma ray emission probability of more than 0.06% per decay (Based on Evaluated Nuclear Structure Data File [4]) was considered. The dose scoring grid was set to 0.1 mm, which was determined by considering the cut-off energy for beta ray. According to the manufacture specification and the previous report, the source shape and size had to be reconstructed on the simulation [5]. The applicator has three layers, which are all made of silver. The thickness of the inner surface, middle layer, and outer surface are 0.1, 0.2, and 0.3 mm, respectively. The radioactive source is stored in the middle layer, with a 0.8 mm peripheral rim that does not contain a radioactive source [5]. The radioactive source was then defined as the uniform distribution. The calculated depth dose distribution was compared to the certification of the depth dose distribution to validate the simulation results. Additionally, according to the previous reports, measuring the applicator’s dose profile was difficult due to difficulty in measuring β-ray precisely, and the Monte Carlo method was considered the most accurate way to calculate the theoretical absorbed dose to water [5, 6]. As a result, the lateral dose profile at a distance of 2.3 mm from the midpoint of the applicator’s inner surface was compared with that reported in previous studies. The simulation had been performed until achieving the uncertainty of the calculated lateral dose distribution at 2.3 mm from the midpoint within 5%, and the uncertainty of the depth dose distribution on the central axis reached less than 5% within 8 mm depth.

### Dose evaluation in treatment geometry

Figure 3 depicts a schematic diagram of the horizontal plane of the patient’s right eye. Despite the fact that the tumor thickness was less than 1 mm, the reference point was set to 1 mm above the surface of the retina including the safety margin. This study focused on four representative locations of the edge of the tumor (Positions 1–4 in Fig. 3) where the dose coverage was expected to be low. Position 1 was defined as 1 mm above the lateral edge of optic sheath. Position 2 was defined as 1 mm above the lateral edge of the tumor. Position 3 was defined as the retinal surface at the lateral edge of the tumor. Position 4 was defined as the retinal surface of the lateral edge of the optic sheath. Additionally, this study also focused on two locations in the optic disc (Positions 5 and 6 in Fig. 3). Position 5 was defined as the retinal surface of the lateral edge of the optic disc. Position 6 was defined as 1 mm depthfrom the retinal surface of the lateral edge of the optic disc. The delivered doses in each location were calculated by changing the distance (*d*) between the edge of the applicator and the eyeball in Fig. 3 to investigate the optimal placement method of the applicator in variable tilting angle to fully cover the tumor as much as possible. The dose distribution was calculated using the Monte Carlo simulation in each case. The acceptance criterion of plaque brachytherapy is to cover the entire tumor with a minimal dose of 40 Gy.

## Results

### Validation of calculated dose distribution

**Fig. 4:**
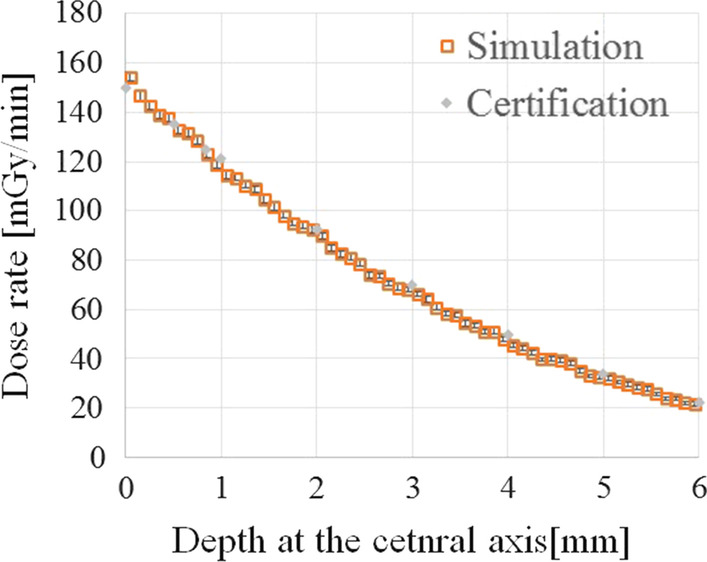
Comparison between the certification and the simulation for the dose rate along the depth direction at the applicator’s central axis

**Fig. 5:**
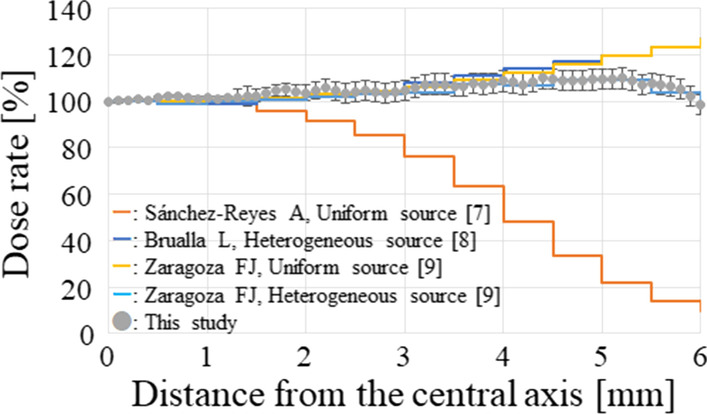
Comparison between this study and the previous reports for the calculated lateral dose profile at a distance of 2.3 mm from the midpoint of the applicator’s inner surface

Figure 4 compares the depth dose rate distributions provided by the certification to those calculated by the simulation. The dose rate discrepancies at a depth of 0.5–6.0 mm were less than 4%. Furthermore, the lateral dose profile at 2.3 mm from the midpoint of the applicator’s inner surface (scoring range: at 2.25–2.35 mm) derived from this study was compared to those at 2.25 mm depth obtained in previous studies (Fig. 5). Despite the difference in radioactive source distribution between the current study and the previous report, the derived lateral dose rate distribution in this study was comparable to that of the previous study [7–9].

### Dose evaluation in treatment geometry

**Fig. 6:**
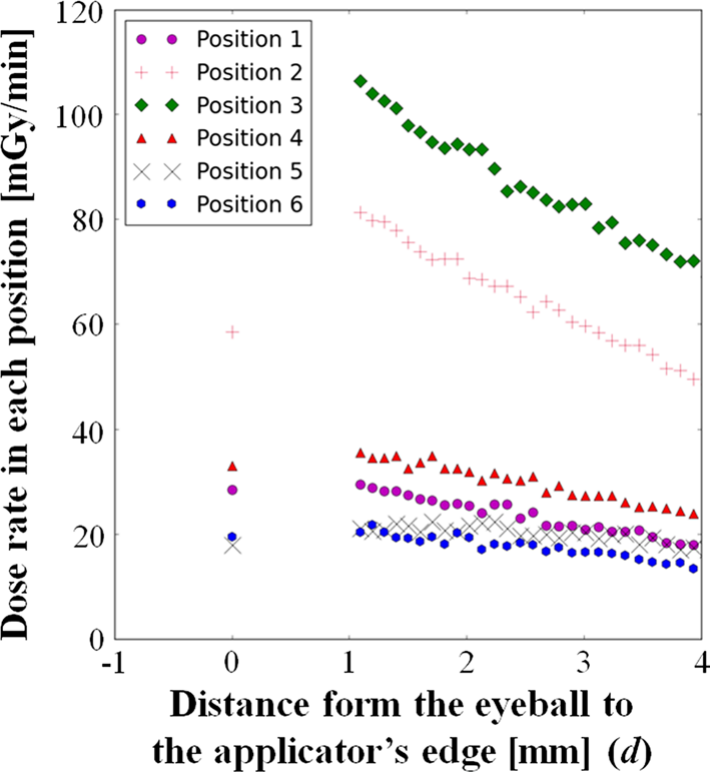
The calculated dose rate in each position. Each of positions was determined as the delivered dose could be low, and its numbers corresponded to Fig. 3. The fixation of the applicator was difficult with the distance from the eyeball to the applicator’s edge between 0 and 1 mm because the applicator edge on the eyeball would float owing to the size difference of the eyeball and the applicator, and the dose rate within this distant range was not then calculated. However, as the representative data, the dose rate at the distance of 0 mm was calculated

**Fig. 7:**
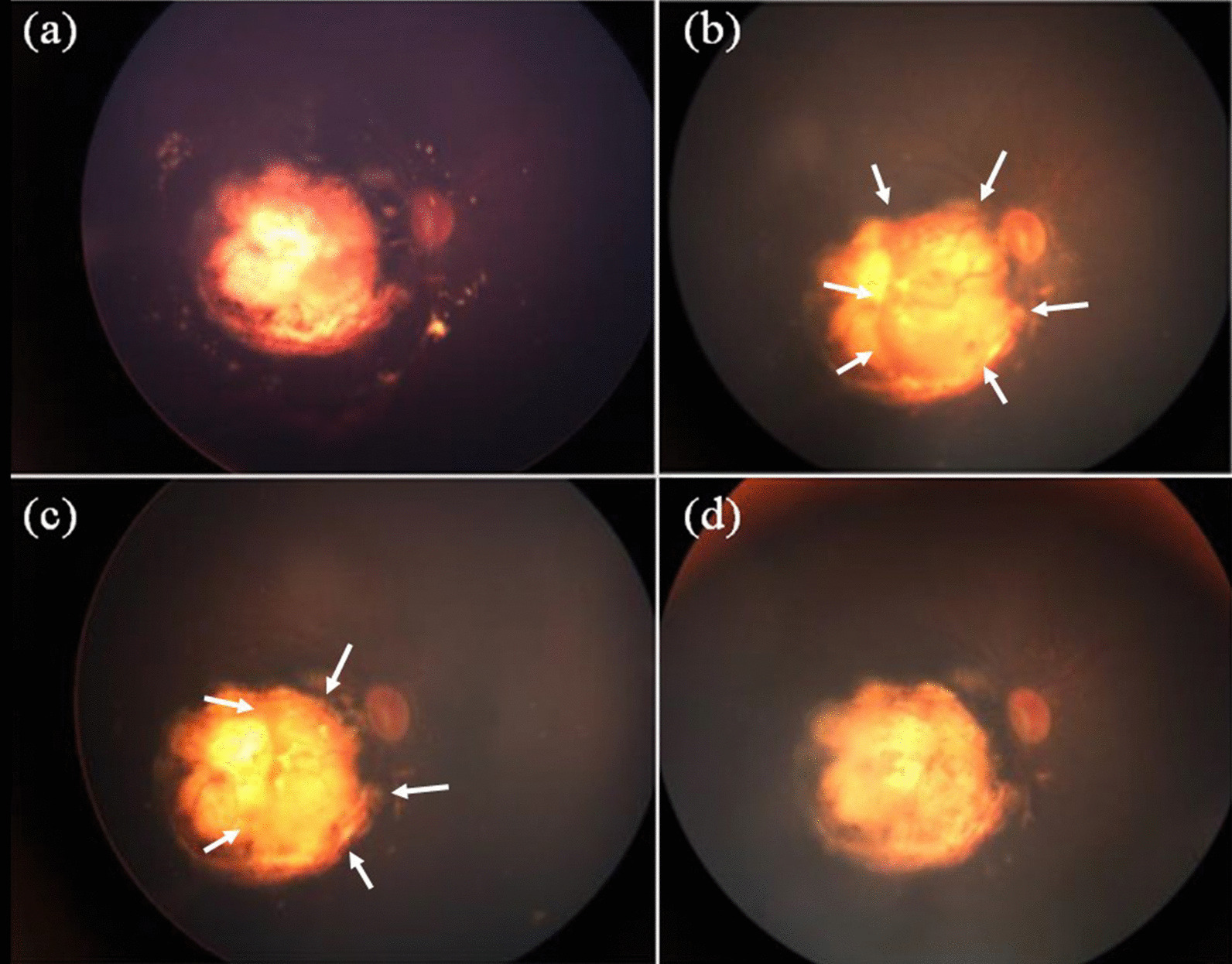
Ocular fundus photographs are shown, **a** just after the previous proton therapy. **b** Five months after the previous proton therapy. Tumor regrowth was noted towards the optic disc side. **c** After the systemic chemotherapy (before this treatment using ^106^Ru applicator). Slight tumor regression was found, but vital component still remained on the optic disc side (arrow). **d** Three months after this treatment. A tumor response was found, and the tumor turned inactive scar-like tissue

Figure 6 shows the results of the calculated dose rate in each position (1–6). According to Fig. 6, the dose at position 1 and 4 were lower than the dose at position 2 and 3. Additionally, when the distance from the eyeball to the applicator’s edge (*d*) varied between 0 and 4 mm, the dose rates among the position 1–4 were higher at 1 mm in almost all cases, and those were then reduced depending on its distance. On the other hand, in positions 5 and 6, the dose rate was gradually increased as *d* became longer (0 ≦ *d* ≦ 2 mm). As mentioned earlier, because the distance (*d*) of equal to or more than 2 mm had to be selected due to the staphyloma, its distance was set 2 mm for the actual treatment to adequately cover the medial and lateral parts of the tumor. In these settings, the minimum dose rate is located at position 1, and its dose rates was 25.5 mGy/min. The required time to deliver the prescribed dose (40 Gy) to position 1 was 26.2 h. When the applicator was attached for the required time, the delivered dose in positions 5 and 6 was 33.6 (21.4 mGy/min) and 30.7 Gy (19.5 mGy/min), respectively. Actually, the applicator was attached for 47.0 h to treat the patient due to logistic reasons: if irradiation time would be longer than 24 h, the next available operation room was the next day. The delivered doses to positions 1, 5, and 6 were 71.9, 60.4, 55.0 Gy, respectively. Figure 7 shows a fundus photograph taken three months after the salvage plaque brachytherapy. The physical examination revealed no active tumors.

## Discussion

It has been reported that if the tumor is localized and does not spread to the optic nerve, an entrance to the brain, organ preservation strategies can be taken without jeopardizing survival. Plaque brachytherapy is a standard treatment for both localized choroidal melanoma and retinoblastoma. When treating an ocular tumor with plaque brachytherapy, the applicator should be temporarily placed so that it covers the entire tumor. One of the major structures that obstacles plaque applicator placement is the optic nerve. According to the American Brachytherapy Society guidelines for plaque brachytherapy, juxtapapillary tumors have a higher rate of local failure [1]. However, the delivered dose around the applicator’s peripheral part is not sufficiently provided by the manufacture certification while the depth dose distribution is provided. Therefore, a detailed evaluation of the dose coverage to those tumors is difficult. The tumor in the case presented in this report was located right next to the optic nerve, which was one of the major obstacles in placing the plaque applicator. Furthermore, because the applicator’s radii are longer than the radius of infants’ eye, the part of the tumor covered by the peripheral part of the applicator receives a lower dose in case of the standard placement. Therefore, we attempted to overcome this fundamental problem by placing the applicator with the peripheral part being touched on the eyeball.

Before brachytherapy, there was a space between the medial edge of the tumor and the optic disc (Fig. 7c). However, when the tumor regrowth was observed before the systemic chemotherapy (Fig. 7b), the tumor’s medial edge reached the lateral edge of the optic disc. Therefore, including the clinical target volume (CTV) on the surface of the optic disc in this study was deemed optimal. According to Fig. 6, the dose rates in positions 5 and 6, which are reference points on the surface of the optic disc, gradually increased as *d* increased from 0 to 2 mm. As a result, the CTV could be covered with a dose that was expected to have therapeutic effect using the method proposed in this study. Therefore, the tilted ^106^Ru plaque applicator placement was one of the appropriate options to covering the dose to tumors located just next to the optic nerve or the optic disc. As mentioned before, although tumors extending to the optic disc should be enucleated because the optic nerve is an entrance to the brain, this tilted applicator placement technique may be able to deliver an adequate dose for tumors extending to the optic disc.

In this study, the calculated lateral dose profile, which applied the uniform radioactive source distribution, was validated by the previously reported calculated results obtained by Monte Carlo simulations. The calculated lateral dose profile was comparable to that of the previous study, which applied the heterogeneous radioactive source distribution in the applicator rather than the uniform radioactive source distribution [7–9]. It could be related to the difference of Monte Carlo simulation code. In the previous report, the difference in dose distributions between different Monte Carlo simulation codes was investigated [5]. It was indicated that the difference observed in the report was due to differences in the geometrical reconstruction ability of the ^106^Ru source between different codes. The PHITS Monte Carlo code was used in this study, whereas the previous studies used GEANT 4. However, previous study recommended that the results of Monte Carlo simulation should be used for guidance the when the uniform radioactive source distribution was assumed or when the dose distribution derived from the Monte Carlo simulation was not sufficiently validated by the dosimetry measurements using the actual applicator [5]. Actually, it appeared that the Monte Carlo simulation in this study did not sufficiently reflect the actual applicator’s source data because the previous study investigated the difference of the dose distributions among plaques between different serial numbers of the same plaque type [10]. Thus, following those recommendations, the simulation results were utilized for the guidance in this study. Previous study also suggested that the lateral dose profile was overestimated when a uniform radioactive source was applied in Monte Carlo simulation [10]. As a result, taking into account the recommendations, the clinical significances (including dose to the CTV), and the logistic reasons, the actual treatment time was longer than the required time (26.2 h). Hence, in this patient, the delivered dose to the optic disc exceeded the threshold dose for blindness. Although there was a possibility of developing blindness due to the late adverse effects of the treatment, there were no other effective treatment options, and blindness was a matter of chance rather than guarantee, so the final decision was made after much deliberation. However, because the dose rate effect of normal tissue and recovery from sublethal damage can be expected, this may not be the case; therefore, long-term follow-up is necessary. Additionally, the optimal placement of the ^106^Ru applicator was calculated by the two-dimensional dose distribution. Thus, the volume effect was not taken into account in this study, which was its limitation.

## Conclusions

This study investigated the tilted ^106^Ru plaque brachytherapy placement to deliver the prescribed dose to an infant with juxtapapillary retinoblastoma when the standard placement was not possible. Optimizing the distance between the edge of the plaque applicator and the eyeball by simulation, the tilted placement for the plaque applicator can promise to deliver the prescribed dose to the tumor even if the tumor is located just next to the optic nerve, which is one of the major obstacles in placing the plaque applicator. Therefore, the favorable treatment outcome can be expected by applying the tilted placement to an infant with juxtapapillary retinoblastoma when the standard plaque placement is difficult.

## Data Availability

ll data generated and/or analysed during this study are included in this published article.

## Abbreviations

ICRB: The International Classification of Retinoblastoma
ICRU: International Commission on Radiation Units and Measurements
CTV: Clinical target volume
PHITS: Particle and Heavy Ion Transport code Systems
